# Multimorbidity and health-related quality of life in Koreans aged 50 or older using KNHANES 2013–2014

**DOI:** 10.1186/s12955-018-1016-6

**Published:** 2018-09-15

**Authors:** Bomi Park, Minsu Ock, Hye Ah Lee, Seonhwa Lee, Hyejin Han, Min-Woo Jo, Hyesook Park

**Affiliations:** 1 Department of preventive medicine, School of Medicine, Ewha Womans University, Seoul, South Korea; 2 Department of Preventive Medicine, Ulsan University Hospital, University of Ulsan College of Medicine, Ulsan, South Korea; 3 Clinical Trial Center, Mokdong Hospital, Ewha Womans University, Seoul, South Korea; 4 Department of Preventive Medicine, University of Ulsan College of Medicine, Seoul, South Korea

**Keywords:** Multimorbidity, Health-related quality of life, Prevalence, Socioeconomic disparity

## Abstract

**Background:**

Multimorbidity negatively affects health outcomes and impairs health-related quality of life (HRQoL). We assessed the prevalence of multimorbidity in Koreans aged 50 and older, taking into consideration their socioeconomic status, and estimated the loss in HRQoL due to multimorbidity.

**Methods:**

This study is based on an analysis of data for adults aged 50 and older derived from the cross-sectional nationally representative Korean National Health and Nutrition Examination Survey conducted in 2013–14. The five most prevalent chronic diseases and disease dyads were identified. The impact of the degree of multimorbidity, sex, and socioeconomic status on the European Quality of Life 5 Dimension (EQ-5D) index score were analyzed. Marital status, educational attainment, household income, basic livelihood security benefit, and occupation were considered as socioeconomic factors.

**Results:**

The analysis included 5996 adults aged 50 years and older with males comprising 46.6%. Two or more chronic diseases were present in 26.8% of the participants aged 50 and older and 37.9% of the participants aged 65 and older. The most prevalent dyadic combination was hypertension and dyslipidemia in the 50 and older group, and hypertension and osteoarthritis in the 65 and older age group. Hypertension dominated the multimorbidity combinations (four of the five most prevalent multimorbidity dyads), while a few conditions such as osteoarthritis had a relatively large influence on quality of life. In addition to the degree of multimorbidity, female and lower socioeconomic status were associated with significantly lower EQ-5D index scores.

**Conclusions:**

Integrated, holistic healthcare based on a patient-oriented perspective for earlier, more effective intervention, targeting multimorbidity is warranted. Special consideration should be given to patients with low socioeconomic status.

## Background

Multimorbidity is defined as the simultaneous presence of two or more diseases in the same individual [1]. With technological advances and improvements in medical care and public policy, chronic medical conditions have overtaken infectious diseases as the dominant cause of death and disability. This phenomenon, in parallel with the aging population, has led to an increase in the number of individuals who present with multimorbidity of chronic diseases [2, 3].

Multimorbidity is important for both patients and the healthcare system, and a great public health concern, because multimorbidity is associated with higher mortality [4, 5], reduced functional status [6], lower quality of life [7], longer hospitalization, higher readmission, more frequent healthcare utilization, and higher healthcare costs [8–10].

Since chronic conditions are controlled rather than cured, the amount of multimorbidity has a large influence on the quality of life until death. Previous studies reported high prevalence rates of multimorbidity in older people ranging from 55 to 98% in different settings, as well as an inverse relationship between multimorbidity and quality of life [2, 11]. However, little is known about socioeconomic inequalities in quality of life in the context of multimorbidity. In addition, only a few Korean studies have focused on multimorbidity, even though Korea is an aged society where an estimated 28.7% of the population will be older than 65 years by 2035 and 42.5% by 2065 [12]. Therefore, this study investigated the prevalence of multimorbidity among Koreans aged 50 or older and explored whether the number of co-occurring chronic conditions in concert with socioeconomic status affect the quality of life of those with multimorbidity.

## Methods

### Data source

This study is based on an analysis of adults aged 50 and older using data derived from the 6th Korean National Health and Nutrition Examination Survey (KNHANES) conducted in 2013–14. KNHANES is a series of cross-sectional national surveys of the noninstitutionalized Korean population administered by the Division of Chronic Disease Surveillance of the Korean Centers for Disease Control and Prevention (KCDC), and consists of a health interview, health examination, and nutrition survey. KNHANES used a complex, stratified, multistage cluster sampling method based on geographical area, gender, and age to secure a representation of the Korean population. Primary sampling units (PSUs) were drawn from a sampling frame of all census blocks or resident registration addresses, and then households were sampled for each PSU using systematic sampling. In the selected households, individuals aged 1 year and over were targeted. This study was approved by the institutional review board of KCDC (2013-07CON-03-4C, 2013-12EXP-03-5C), and written informed consent to participate in the study was obtained from all participants.

### Chronic diseases

Multimorbidity was defined as having two or more of 25 chronic diseases pre-specified in KNHANES: hypertension, dyslipidemia, stroke, myocardial infarction, angina, osteoarthritis, rheumatic arthritis, tuberculosis, asthma, allergic rhinitis, depression, kidney failure, atopic dermatitis, diabetes mellitus, thyroid disease, stomach cancer, liver cancer, colon cancer, breast cancer, cervical cancer, lung cancer, thyroid cancer, hepatitis B, hepatitis C, and liver cirrhosis. The presence of disease was determined based on self-reports of whether the participants had ever been diagnosed with each disease by a physician and either currently had that disease or were under treatment for that disease.

### Health related quality of life

Health related quality of life (HRQoL) was assessed using the EuroQol 5 Dimensions (EQ-5D), a generic measure of HRQoL that can be used to describe and value health states. The EQ-5D consists of five dimensions (mobility, self-care, usual activities, pain or discomfort, and anxiety or depression) with three response levels for each dimension (no problem, moderate problem, and severe problem) generating a total of 243 different health states. The EQ-5D produces a single utility value of a given health state on a scale where 0 indicates death, 1 indicates perfect health, and negative values indicate health states worse than death. The utility value was transformed from the EQ-5D response using the Korean EQ-5D-3 L-value set [13].

### Socioeconomic factors

We considered the following socioeconomic factors: marital status, educational attainment, household income, basic livelihood security benefit, and occupation. Marital status was divided into two categories: married and unmarried (single, separated, divorced, or widowed). Educational attainment was categorized into two groups. A high school degree or less was defined as low educational attainment for those aged less than 70, while a middle school degree or less was defined as low for those aged 70 years and older. The household income was calculated by dividing the household monthly income by the square root of the household size [14], and then categorized into quartiles, with 1 being the lowest and 4 being the highest. The cut-off values for quartiles were 667.70, 1335.41, and 2192.82 US dollars in 2013 and 605.92, 1376.96, and 2225.68 US dollars in 2014. Occupation status was categorized into unemployed, blue-collar worker, and white-collar worker. Blue-collar worker includes service workers or retailers, agriculture or fishery employees, technicians, mechanics, or assemblers, and simple laborers. White-collar worker includes managers, professionals, and office workers.

### Statistical analysis

Descriptive analysis was performed for the participants’ socioeconomic and clinical characteristics. Because of the non-normal distribution of the EQ-5D index score, it was log-transformed before further analysis and the EQ-5D index scores are presented as geometric means with the 95% confidence intervals (CIs) after back-transformation. All estimates were weighted to account for the complex survey design, so that the results would be representative of the entire Korean population. Participants’ sociodemographic and clinical characteristics are presented as the sample-weighted percentage of the population with the standard error (SE) or the sample-weighted geometric mean with the 95% CI. The percentage of multimorbidity and EQ-5D index score across socioeconomic status was estimated. The five most prevalent chronic diseases and disease dyads were identified within each age group and stratified by sex, and the prevalence rate was defined as the total number of cases divided by the average registered population of Korean residents in 2013 and 2014.

Estimates of the least square means for EQ-5D index score with 95% CIs by the number of chronic conditions and socioeconomic status (marital status, educational attainment, household income, basic livelihood security benefit, and occupation) were calculated using regression analysis with SAS PROC SURVEYREG. In an adjusted model, least square means for EQ-5D index score with 95% CIs by the number of chronic conditions were adjusted for marital status, education, household income, basic livelihood security recipient status, and occupation. Sex was additionally adjusted in analyses with the total population, including both sexes.

All analyses were repeated with only participants aged 65 and older to specifically investigate the aged population. All tests were two-tailed, and a *p*-value < 0.05 was regarded as statistically significant. All statistical analyses were performed using SAS (ver. 9.4; SAS Institute, Cary, NC, USA) with the survey procedure.

## Results

The sample consisted of 5996 (weighted frequency 16,591,498) participants aged 50 years and older (46.6% male) and 2856 (weighted frequency 6,261,631) participants aged 65 years and older (41.5% male). The mean age of the study population was 62.7 (95% CI 62.3–63.0) years. Table 1 presents the sociodemographic and clinical characteristics according to age group and sex.

**Table 1:** Participants’ sociodemographic and clinical characteristics by gender

Variable	50 and older				65 and older			
	Total	Male	Female	*p*-value	Total	Male	Female	*p*-value
Number of chronic conditions								
0	46.6 (0.9)	52.4 (1.2)	41.5 (1.1)	< 0.0001	32.9 (1.2)	38.7 (1.7)	28.7 (1.5)	< 0.0001
1	26.6 (0.7)	27.2 (1.1)	26.1 (0.9)		29.2 (1.1)	32.9 (1.6)	26.5 (1.3)	
2	15.7 (0.6)	12.7 (0.8)	18.4 (0.8)		20.3 (0.9)	17.4 (1.2)	22.4 (1.2)	
3	7.0 (0.4)	5.1 (0.5)	8.7 (0.6)		10.8 (0.7)	7.3 (0.9)	13.2 (1.0)	
4+	4.0 (0.3)	2.6 (0.3)	5.3 (0.5)		6.9 (0.6)	3.7 (0.6)	9.1 (0.9)	
EQ-5D index score	0.89 (0.89–0.90)	0.93 (0.92–0.93)	0.87 (0.86–0.88)	< 0.0001	0.83 (0.82–0.84)	0.88 (0.86–0.90)	0.79 (0.78–0.81)	< 0.0001
Current marital status								
Married	76.8 (0.8)	88.5 (0.9)	66.5 (1.0)	< 0.0001	62.1 (1.2)	88.1 (1.1)	43.7 (1.4)	< 0.0001
Unmarried	23.2 (0.8)	11.5 (0.9)	33.5 (1.0)		37.9 (1.2)	11.9 (1.1)	56.3 (1.4)	
Education^a^								
High	17.8 (0.9)	26.8 (1.3)	10.1 (0.8)	< 0.0001	15.7 (1.0)	28.0 (1.7)	6.8 (0.8)	< 0.0001
Low	82.2 (0.9)	73.2 (1.3)	89.9 (0.8)		84.3 (1.0)	72.0 (1.7)	93.2 (0.8)	
Household income^b^								
Quartile 4	23.8 (1.1)	26.3 (1.3)	21.6 (1.1)	< 0.0001	9.9 (0.8)	11.9 (1.2)	8.4 (0.9)	< 0.0001
Quartile 3	22.3 (0.8)	24.6 (1.0)	20.3 (0.9)		13.2 (0.9)	15.3 (1.2)	11.7 (1.0)	
Quartile 2	25.8 (0.9)	26.6 (1.1)	25.1 (1.0)		25.9 (1.2)	29.0 (1.6)	23.7 (1.2)	
Quartile 1	28.1 (1.1)	22.5 (1.1)	33.0 (1.3)		51.0 (1.6)	43.7 (1.9)	56.2 (1.7)	
Basic livelihood security recipients								
No	94.6 (0.6)	95.5 (0.7)	93.9 (0.7)	0.01	91.5 (1.0)	94.0 (1.0)	89.7 (1.2)	0.0004
Yes	5.4 (0.6)	4.5 (0.7)	6.1 (0.7)		8.5 (1.0)	6.0 (1.0)	10.3 (1.2)	
Occupation								
White-collar	10.9 (0.7)	17.1 (1.1)	5.5 (0.5)	< 0.0001	2.9 (0.4)	6.0 (1.0)	0.6 (0.2)	< 0.0001
Blue-collar	42.3 (1.0)	50.3 (1.5)	35.4 (1.1)		27.3 (1.2)	33.1 (1.9)	23.1 (1.3)	
Unemployed	46.8 (1.0)	32.5 (1.3)	59.1 (1.1)		69.8 (1.3)	60.9 (1.9)	76.2 (1.3)	

Values are presented as % of population (standard error) or geometric mean (95% confidence interval)

There was some overlap in the population between the two age groups with different age cut-off values

EQ-5D, European Quality of Life 5 Dimension

^a^Education: low: High school or under for 50–69 years of age and middle school or under for 70 and older years of age; high: college or higher for 50–69 years of age and high school or higher for 70 and older years of age

^b^Household income: household monthly income/$\sqrt{\mathrm{the}\ \mathrm{household}\ \mathrm{size}}$

Among the participants aged 50 and older, 26.8% (SE, 0.8) reported having two or more chronic diseases and 11.0% (SE, 0.5) reported having three or more chronic diseases from the 25-item list. In addition, 37.9% (SE, 1.3) of the Koreans aged 65 and older reported having two or more chronic diseases and 17.6% (SE, 0.9) reported having three or more chronic diseases from the list.

The geometric mean EQ-5D index score was 0.89 (95% CI, 0.89–0.90) for 50 and older, and 0.83 (95% CI, 0.82–0.84) for 65 and older. The EQ-5D index score was significantly lower among women (*p* < 0.0001) for both age groups (Table 1).

Of those aged 50 and older, the multimorbidity percentage was significantly higher and the EQ-5D index scores were significantly lower in those who are currently single, those who attained a lower education level, those living in the lowest house income quartile, those who received basic livelihood security, and those who are unemployed. The same pattern was seen in the 65 and older age group (Table 2).

**Table 2:** Presence of multimorbidity and EQ-5D index score by sociodemographic characteristics

Variable	50 and older		65 and older	
	% of multimorbidity	EQ-5D index score	% of multimorbidity	EQ-5D index score
	(SE)	Mean (95% CI)	(SE)	Mean (95% CI)
Current marital status				
Married	24.1 (0.8)	0.92 (0.91–0.92)	35.7 (1.4)	0.86 (0.85–0.88)
Unmarried	35.7 (1.6)	0.81 (0.80–0.83)	41.8 (2.0)	0.78 (0.75–0.80)
Education^a^				
High	22.4 (1.7)	0.95 (0.94–0.96)	38.0 (2.8)	0.92 (0.90–0.94)
Low	31.3 (0.9)	0.88 (0.87–0.89)	43.7 (1.5)	0.82 (0.80–0.83)
Household income^b^				
Quartile 4	19.6 (1.4)	0.95 (0.94–0.96)	37.1 (3.5)	0.90 (0.88–0.93)
Quartile 3	23.5 (1.4)	0.93 (0.93–0.94)	38.6 (2.8)	0.88 (0.86–0.90)
Quartile 2	26.6 (1.2)	0.90 (0.89–0.91)	37.0 (1.9)	0.86 (0.83–0.88)
Quartile 1	35.9 (1.5)	0.81 (0.79–0.83)	38.7 (1.7)	0.79 (0.77–0.81)
Basic livelihood security recipients				
No	25.9 (0.8)	0.90 (0.90–0.91)	36.9 (1.3)	0.85 (0.83–0.86)
Yes	42.5 (2.8)	0.72 (0.67–0.76)	50.0 (3.5)	0.67 (0.61–0.74)
Occupation				
White-collar	14.7 (1.9)	0.97 (0.96–0.97)	26.9 (6.0)	0.95 (0.92–0.97)
Blue-collar	23.0 (1.1)	0.93 (0.92–0.94)	34.8 (2.1)	0.88 (0.86–0.89)
Unemployed	39.3 (1.1)	0.84 (0.83–0.86)	46.5 (1.5)	0.81 (0.79–0.83)

*N* Number, *SE* Standard Error, *EQ-5D* European Quality of Life 5 Dimension, *CI* Confidence Interval

^a^Education: low: High school or under for 50–69 years of age and middle school or under for 70 and older years of age; high: college or higher for 50–69 years of age and high school or higher for 70 and older years of age

^b^Household income: household monthly income/$\sqrt{\mathrm{the}\ \mathrm{household}\ \mathrm{size}}$

Table 3 shows the five most prevalent chronic diseases and their EQ-5D index scores when the participants reported having only one disease. Among the participants aged 50 and older, the five most prevalent diseases were hypertension (107.55 per 1000 people), osteoarthritis (47.24 per 1000 people), diabetes mellitus (25.21 per 1000 people), dyslipidemia (23.23 per 1000 people), and allergic rhinitis (20.72 per 1000 people). The participants aged 65 and older showed a similar pattern, except that depression was the fifth most prevalent disease, rather than allergic rhinitis.

**Table 3:** Prevalence and EQ-5D index score of the 5 most prevalent chronic conditions

50 and older			65 and older		
Disease	Prevalence (per 1000)	EQ-5D index score (95% CI)	Disease	Prevalence (per 1000)	EQ-5D index score (95% CI)
All					
Hypertension	107.55	0.92 (0.90–0.93)	Hypertension	151.41	0.88 (0.86–0.90)
Osteoarthritis	47.24	0.83 (0.80–0.86)	Osteoarthritis	58.21	0.78 (0.73–0.83)
Diabetes mellitus	25.21	0.92 (0.90–0.95)	Diabetes mellitus	27.08	0.91 (0.87–0.96)
Dyslipidemia	23.23	0.92 (0.90–0.94)	Dyslipidemia	18.23	0.87 (0.81–0.93)
Allergic rhinitis	20.72	0.95 (0.94–0.97)	Depression	6.25	0.85 (0.83–0.88)
Male					
Hypertension	121.24	0.93 (0.91–0.95)	Hypertension	183.70	0.90 (0.87–0.94)
Diabetes mellitus	39.49	0.94 (0.92–0.96)	Diabetes mellitus	47.46	0.94 (0.90–0.98)
Osteoarthritis	26.96	0.85 (0.79–0.92)	Osteoarthritis	43.64	0.82 (0.73–0.92)
Allergic rhinitis	25.83	0.97 (0.95–0.99)	Allergic rhinitis	12.08	0.94 (0.89–1.00)
Dyslipidemia	15.85	0.93 (0.88–0.98)	Dyslipidemia	11.25	0.83 (0.65–1.05)
Female					
Hypertension	95.51	0.90 (0.88–0.92)	Hypertension	128.49	0.86 (0.83–0.89)
Osteoarthritis	65.08	0.83 (0.79–0.86)	Osteoarthritis	68.55	0.76 (0.70–0.82)
Dyslipidemia	29.72	0.91 (0.89–0.94)	Dyslipidemia	23.18	0.89 (0.85–0.93)
Allergic rhinitis	16.22	0.93 (0.90–0.96)	Diabetes mellitus	12.61	0.84 (0.72–0.99)
Diabetes mellitus	12.66	0.87 (0.80–0.94)	Depression	7.53	0.82 (0.80–0.83)

*EQ-5D* European Quality of Life 5 Dimension, *CI* Confidence Interval

EQ-5D index score was presented as geometric mean (95% CI)

There were no differences between men and women in the lists of the five most prevalent diseases, but the rank order of the lists differed. Men had higher prevalences of hypertension (121.24 per 1000 vs. 95.41 per 1000), diabetes mellitus (39.49 per 1000 vs. 12.66 per 1000), and allergic rhinitis (25.83 per 1000 vs. 16.22 per 1000), while women had higher prevalences of osteoarthritis (65.08 vs. 26.96 per 1000) and dyslipidemia (29.72 vs. 15.85 per 1000) (Table 3).

Table 4 shows the five most prevalent dyadic combinations and their EQ-5D index scores. The disease dyads most commonly observed among the 50 and older age group were hypertension and dyslipidemia (27.89 per 1000), followed by hypertension and diabetes mellitus (25.14 per 1000), while the 65 and older age group experienced the combination of hypertension and osteoarthritis the most (42.73 per 1000), followed by hypertension and diabetes mellitus (42.50 per 1000). The most frequent individual chronic disease, hypertension, dominated the dyadic combinations and was present in four out of the top five prevalent dyads for both age groups.

**Table 4:** Prevalence and EQ-5D index score of the 5 most prevalent chronic disease dyads

50 and older			65 and older		
Disease combination	Prevalence (per 1000)	EQ-5D index score (95% CI)	Disease combination	Prevalence (per 1000)	EQ-5D index score (95% CI)
All					
Hypertension and Dyslipidemia	27.89	0.93 (0.91–0.95)	Hypertension and Osteoarthritis	42.73	0.78 (0.74–0.82)
Hypertension and Diabetes mellitus	25.14	0.87 (0.82–0.93)	Hypertension and Diabetes mellitus	42.50	0.84 (0.76–0.92)
Hypertension and Osteoarthritis	23.35	0.81 (0.77–0.85)	Hypertension and Dyslipidemia	28.05	0.93 (0.91–0.96)
Dyslipidemia and Diabetes mellitus	7.61	0.94 (0.90–0.98)	Osteoarthritis and Diabetes mellitus	7.39	0.79 (0.72–0.87)
Hypertension and Allergic rhinitis	6.57	0.95 (0.92–0.97)	Hypertension and Stroke	7.35	0.72 (0.61–0.86)
Male					
Hypertension and Diabetes mellitus	30.04	0.86 (0.77–0.96)	Hypertension and Diabetes mellitus	52.80	0.82 (0.69–0.98)
Hypertension and Dyslipidemia	23.25	0.94 (0.91–0.98)	Hypertension and Dyslipidemia	27.92	0.96 (0.92–0.99)
Dyslipidemia and Diabetes mellitus	8.90	0.96 (0.95–0.98)	Hypertension and Osteoarthritis	16.33	0.80 (0.66–0.97)
Hypertension and Osteoarthritis	8.09	0.82 (0.71–0.94)	Hypertension and Stroke	7.95	0.86 (0.79–0.93)
Hypertension and Allergic rhinitis	7.77	0.94 (0.88–1.01)	Hypertension and Angina	6.06	0.95 (0.77–1.17)
Female					
Hypertension and Osteoarthritis	36.76	0.81 (0.77–0.85)	Hypertension and Osteoarthritis	61.46	0.77 (0.73–0.81)
Hypertension and Dyslipidemia	31.96	0.92 (0.90–0.95)	Hypertension and Diabetes mellitus	35.19	0.86 (0.83–0.89)
Hypertension and Diabetes mellitus	20.82	0.89 (0.86–0.92)	Hypertension and Dyslipidemia	28.14	0.92 (0.89–0.95)
Dyslipidemia and Osteoarthritis	10.41	0.86 (0.83–0.89)	Osteoarthritis and Diabetes mellitus	11.73	0.79 (0.71–0.88)
Osteoarthritis and Allergic rhinitis	8.02	0.88 (0.83–0.94)	Dyslipidemia and Osteoarthritis	8.71	0.85 (0.81–0.89)

EQ-5D index score was presented as geometric mean (95% CI)

Hypertension was the most frequent single chronic disease, but the EQ-5D index score was the lowest for osteoarthritis in both age groups and both sexes (Table 3), and the combination of hypertension and osteoarthritis had the lowest EQ-5D index score out of the top five most prevalent dyads for both men and women (Table 4).

The associations between the number of chronic diseases or socioeconomic status with the EQ-5D index score were analyzed, and the results are summarized in Table 5. The least square mean of EQ-5D index score for those who had more chronic diseases was significantly lower compared with the reference group for both age groups and sex. Regarding socioeconomic status, unmarried status, lower education level, lower household income, basic livelihood security recipient status, and unemployed status were associated with a lower EQ-5D index score (Table 5). When we considered sex, the number of chronic conditions and socioeconomic status together in one model, female, greater number of chronic conditions, and lower socioeconomic status were still significantly associated with a lower EQ-5D index score (Table 6).

**Table 5:** Least square means of EQ-5D index score by the degree of multimorbidity and socioeconomic status

	50 and older			65 and older		
	Total	Male	Female	Total	Male	Female
Number of chronic conditions						
0 (reference)	0.94 (0.93–0.95)	0.96 (0.95–0.96)	0.92 (0.91–0.93)	0.88 (0.86–0.90)	0.93 (0.91–0.95)	0.83 (0.79–0.86)
1	0.90 (0.89–0.91)*	0.92 (0.91–0.94)*	0.88 (0.86–0.89)*	0.86 (0.84–0.88)	0.89 (0.87–0.92)*	0.83 (0.80–0.85)
2	0.87 (0.85–0.88)*	0.90 (0.87–0.92)*	0.85 (0.84–0.87)*	0.83 (0.80–0.85)*	0.86 (0.82–0.91)*	0.81 (0.78–0.84)
3	0.81 (0.78–0.84)*	0.84 (0.79–0.90)*	0.79 (0.76–0.82)*	0.77 (0.73–0.82)*	0.80 (0.70–0.91)*	0.76 (0.72–0.80)*
4+	0.71 (0.67–0.75)*	0.78 (0.72–0.84)*	0.68 (0.63–0.73)*	0.69 (0.64–0.74)*	0.76 (0.68–0.84)*	0.67 (0.61–0.73)*
Marital status						
Married (reference)	0.92 (0.91–0.92)	0.93 (0.92–0.94)	0.90 (0.89–0.91)	0.86 (0.85–0.88)	0.89 (0.87–0.91)	0.83 (0.81–0.85)
Unmarried	0.81 (0.80–0.83)*	0.87 (0.84–0.90)*	0.80 (0.78–0.82)*	0.78 (0.75–0.80)*	0.85 (0.81–0.90)	0.77 (0.74–0.79)*
Education						
High (reference)	0.95 (0.94–0.96)	0.95 (0.94–0.96)	0.94 (0.93–0.96)	0.92 (0.90–0.94)	0.92 (0.91–0.94)	0.91 (0.88–0.94)
Low	0.88 (0.87–0.89)*	0.92 (0.91–0.93)*	0.86 (0.85–0.87)*	0.82 (0.80–0.83)*	0.87 (0.85–0.89)*	0.79 (0.77–0.81)*
Household income						
Quartile 4 (reference)	0.95 (0.94–0.96)	0.97 (0.96–0.98)	0.93 (0.92–0.94)	0.90 (0.88–0.93)	0.94 (0.92–0.96)	0.87 (0.83–0.91)
Quartile 3	0.93 (0.93–0.94)*	0.96 (0.95–0.96)*	0.91 (0.90–0.92)*	0.88 (0.86–0.90)	0.92 (0.90–0.94)	0.84 (0.81–0.87)
Quartile 2	0.90 (0.89–0.91)*	0.93 (0.91–0.94)*	0.87 (0.86–0.89)*	0.86 (0.83–0.90)*	0.90 (0.87–0.94)	0.82 (0.79–0.84)*
Quartile 1	0.81 (0.79–0.83)*	0.84 (0.82–0.87)*	0.79 (0.77–0.81)*	0.79 (0.77–0.81)*	0.84 (0.81–0.87)*	0.76 (0.74–0.79)*
Basic livelihood security recipients						
No (reference)	0.90 (0.90–0.91)	0.93 (0.92–0.94)	0.88 (0.87–0.89)	0.85 (0.83–0.86)	0.89 (0.87–0.91)	0.81 (0.80–0.83)
Yes	0.72 (0.68–0.76)*	0.78 (0.73–0.83)*	0.68 (0.62–0.75)*	0.67 (0.61–0.74)*	0.79 (0.71–0.87)*	0.63 (0.56–0.72)*
Occupation						
White-collar (reference)	0.97 (0.96–0.97)	0.97 (0.97–0.98)	0.95 (0.94–0.97)	0.95 (0.92–0.97)	0.95 (0.92–0.97)	0.93 (0.88–0.99)
Blue-collar	0.93 (0.92–0.94)*	0.95 (0.95–0.96)*	0.91 (0.89–0.92)*	0.88 (0.86–0.89)*	0.92 (0.90–0.93)*	0.84 (0.82–0.86)*
Unemployed	0.84 (0.83–0.86)*	0.86 (0.84–0.89)*	0.84 (0.82–0.85)*	0.81 (0.79–0.83)*	0.86 (0.83–0.89)*	0.78 (0.76–0.80)*

EQ-5D index score was presented as geometric mean (95% CI)

*p < 0.05 compared to reference group

**Table 6:** Adjusted least square means of EQ-5D index score by the degree of multimorbidity

	50 and older			65 and older		
	Total	Male	Female	Total	Male	Female
Sex						
Male (Reference)	0.82 (0.80–0.85)			0.81 (0.77–0.86)		
Female	0.81 (0.78–0.83)*			0.79 (0.74–0.83)*		
Number of chronic conditions						
0 (reference)	0.88 (0.85–0.91)	0.91 (0.88–0.94)	0.86 (0.82–0.90)	0.86 (0.81–0.91)	0.94 (0.88–0.99)	0.83 (0.77–0.90)
1	0.86 (0.83–0.88)*	0.89 (0.86–0.92)*	0.84 (0.81–0.88)*	0.85 (0.80–0.89)	0.90 (0.85–0.96)*	0.84 (0.79–0.90)
2	0.84 (0.82–0.87)*	0.87 (0.83–0.90)*	0.83 (0.80–0.87)*	0.82 (0.78–0.86)*	0.87 (0.81–0.93)*	0.82 (0.76–0.88)
3	0.80 (0.76–0.83)*	0.83 (0.77–0.89)*	0.78 (0.74–0.83)*	0.78 (0.73–0.84)*	0.82 (0.72–0.93)*	0.79 (0.73–0.86)
4+	0.71 (0.66–0.75)*	0.76 (0.71–0.82)*	0.68 (0.63–0.74)*	0.70 (0.64–0.76)*	0.77 (0.67–0.88)*	0.69 (0.62–0.77)*
Marital status						
Married (reference)	0.83 (0.81–0.86)	0.86 (0.83–0.88)	0.82 (0.78–0.86)	0.81 (0.77–0.85)	0.86 (0.81–0.91)	0.81 (0.76–0.87)
Unmarried	0.80 (0.77–0.82)*	0.84 (0.80–0.88)	0.78 (0.74–0.82)*	0.79 (0.74–0.83)*	0.86 (0.79–0.93)	0.77 (0.72–0.83)*
Education						
High (reference)	0.83 (0.80–0.86)	0.86 (0.83–0.89)	0.82 (0.78–0.86)	0.83 (0.79–0.88)	0.88 (0.82–0.95)	0.84 (0.78–0.90)
Low	0.80 (0.77–0.82)*	0.84 (0.81–0.86)*	0.78 (0.74–0.82)*	0.77 (0.73–0.81)*	0.83 (0.78–0.89)*	0.75 (0.70–0.80)*
Household income						
Quartile 4 (reference)	0.84 (0.81–0.86)	0.87 (0.84–0.90)	0.82 (0.78–0.86)	0.83 (0.78–0.88)	0.88 (0.83–0.95)	0.82 (0.76–0.89)
Quartile 3	0.83 (0.81–0.86)	0.87 (0.84–0.90)	0.82 (0.78–0.86)	0.81 (0.77–0.86)	0.87 (0.82–0.94)	0.80 (0.74–0.87)
Quartile 2	0.81 (0.79–0.84)*	0.85 (0.82–0.89)*	0.79 (0.76–0.84)*	0.79 (0.75–0.84)*	0.86 (0.79–0.93)	0.78 (0.72–0.84)*
Quartile 1	0.77 (0.75–0.80)*	0.81 (0.78–0.84)*	0.76 (0.72–0.79)*	0.77 (0.73–0.81)*	0.81 (0.76–0.87)*	0.76 (0.71–0.82)*
Basic livelihood security recipients						
No (reference)	0.86 (0.85–0.88)	0.89 (0.86–0.91)	0.86 (0.84–0.87)	0.87 (0.85–0.88)	0.88 (0.85–0.92)	0.87 (0.85–0.90)
Yes	0.77 (0.72–0.81)*	0.81 (0.77–0.86)*	0.74 (0.68–0.81)*	0.74 (0.67–0.81)*	0.83 (0.74–0.93)	0.72 (0.63–0.81)*
Occupation						
White-collar (reference)	0.82 (0.79–0.85)	0.86 (0.83–0.89)	0.80 (0.76–0.85)	0.81 (0.76–0.86)	0.87 (0.81–0.93)	0.81 (0.74–0.89)
Blue-collar	0.83 (0.81–0.86)*	0.87 (0.84–0.90)	0.81 (0.78–0.85)	0.82 (0.78–0.86)	0.88 (0.81–0.94)	0.81 (0.75–0.86)
Unemployed	0.79 (0.77–0.82)*	0.82 (0.79–0.89)*	0.78 (0.74–0.81)*	0.77 (0.74–0.81)*	0.83 (0.78–0.89)	0.76 (0.71–0.81)*

Adjusted for marital status, education, household income, basic livelihood security recipient status, and occupation

Sex was additionally adjusted when analyzed with total including both sex

*p < 0.05 compared to reference group

## Discussion

This study found that the degree of multimorbidity was higher in the older age group. More than one-fourth of the population aged 50 years and older have at least two chronic conditions at once and nearly two out of five elderly aged 65 years and older live with multimorbidity, suggesting that multimorbidity is a common problem in Korean elderly.

Quality of life as measured by the EQ-5D index decreases significantly as the number of comorbid medical conditions increases. Furthermore, a few conditions, such as osteoarthritis, had a larger influence on the quality of life, which suggests that managing key chronic diseases to improve quality of life in the older population is important. We also found that lower socioeconomic status was associated with more prevalent multimorbidity and a lower EQ-5D index score.

With the aging of the population, the prevalence of chronic diseases is increasing and the chance of experiencing co-occurrence of multiple chronic diseases among the elderly population is increasing accordingly. The socioeconomic and disease burdens of multimorbidity are expected to increase considerably due to the rapidly aging population, because patients with multimorbidity are more likely to experience adverse health outcomes, frequent health services, and higher medical costs [4, 5, 8–10]. Therefore, multimorbidity is a very important public health issue that needs to be addressed.

The prevalence of multimorbidity seen in our results is in line with other studies, the majority of which reported prevalences of multimorbidity of 20–60%, compared with 26.8% for age 50 and older and 37.9% for age 65 and older in our study [15–21]. The variation in the estimated prevalence of multimorbidity is likely to be due to differences in the set of diseases included, how multimorbidity is defined, population characteristics included, clinical setting, and database used [22].

Our results regarding the epidemiology of multimorbidity highlight the need to face the challenge of multimorbidity. Because multimorbidity is becoming the norm for individuals living with chronic diseases, the traditional disease-oriented approach may be increasingly fragmented, ineffective, and inefficient for patients with chronic diseases. Health care services and policies for chronic diseases, especially in the elderly, have to be developed and implemented within the context of multimorbidity. Integrated and holistic healthcare based on a patient-oriented perspective with a greater awareness of multimorbidity is more appropriate than the traditional disease-oriented approach.

We found that hypertension, dyslipidemia, diabetes mellitus, osteoarthritis, allergic rhinitis, and stroke were the individual conditions that constituted the most frequent dyadic combinations. Another study reported combinations of hypertension, lipid disorder, chronic ischemic heart disease, diabetes mellitus, osteoarthritis, and low back pain as the most frequent multimorbidity [21, 23].

The fact that a small number of chronic diseases dominates across the wide number of variants of frequent multimorbidity suggests that one disease may increase the risk of other diseases. For example, hypertension is a known risk factor for cardiovascular disease [24, 25]. Some clusters of specific diseases may share common pathological pathways. In other words, multimorbidity may not be a matter of random chance, but there is a pattern of disease clustering. If that is the case, a future assessment of the patterns of multimorbidity should provide essential information on the possible presence of chronic diseases known to co-occur, and enable active integrated and holistic prevention and management efforts to deal with multimorbidity more effectively.

There is an inverse relationship between the number of chronic conditions and HRQoL [26–29], and this was confirmed in our study. HRQoL is a measure of health status used to assess the medical effectiveness of interventions and to support public health planning, and the EQ-5D is a generic instrument for comparing HRQoL between populations with different conditions [30]. Because chronic diseases are long-lasting and generally cannot be cured, and may contribute to the worsening of health and social outcomes [2], managing persons with multimorbidity to achieve a better HRQoL for the rest of their lives is important.

Other studies have found socioeconomic inequalities linked to the prevalence of multimorbidity [31–33]. We found that there is not only a higher prevalence of multimorbidity but also a lower HRQoL among persons with lower socioeconomic status. These results suggest that socioeconomic deprivation plays a role in the development of multimorbidity. Multimorbidity itself also has negative socioeconomic impacts, as it increases the frequency and duration of health services utilization and increases healthcare costs, as mentioned above. As a result, the burden of multimorbidity has a greater influence on groups with lower socioeconomic status and the socioeconomic and health inequality becomes even greater. Therefore, special consideration should be given to patients with low socioeconomic status, with multisector efforts aimed at addressing a range of preventable socioeconomic and behavioral determinants and healthcare costs, thus enhancing health outcomes and quality of life.

The strengths of our study include the relatively large study population, which is representative of the general population of Korea, and that it includes data on quality of life and socioeconomic status. However, some limitations should be acknowledged. The results depend partly on the operational definition of multimorbidity used and the number and type of chronic diseases considered [34]. Fortin et al. reported that if more diseases are considered, a higher prevalence will be estimated [22]. According to Harrisson et al. [35], however, including the 12 most prevalent chronic conditions is sufficient to provide reasonable prevalence estimates when defining multimorbidity as having two or more chronic diseases. We considered 25 chronic diseases, limited to those surveyed by KNHANES, which is a relatively long list of chronic conditions; however, future studies covering the entire spectrum of chronic disease may be required. Our results are likely to be subject to recall bias, since the chronic diseases were based on self-reports, and selection bias, as only the non-institutionalized Korean population was included in the survey. However, we expect that the direction of these biases is toward the null and the real effect of multimorbidity would be greater than determined here.

## Conclusions

There is a high prevalence of multimorbidity and lower HRQoL in Korean adults experiencing multimorbidity, especially the female elderly with lower socioeconomic status. Taking into consideration the large disease and economic burden of multimorbidity and its negative effects on HRQoL, the development of appropriate guidelines for the integrated, co-operative management of multiple chronic diseases is warranted. We expect the results of this study to be used as an evidence base to devise informed healthcare for the effective care of patients with multimorbidity.

## Abbreviations

CI: Confidence interval
EQ-5D: EuroQol five-dimension
HRQoL: Health-related quality of life
KCDC: Korean Centers for Disease Control and Prevention
KNHANES: Korean National Health and Nutrition Examination Survey
SE: Standard error
